# Pre-clinical evaluation of EC1456, a folate-tubulysin anti-cancer therapeutic

**DOI:** 10.1038/s41598-018-27320-5

**Published:** 2018-06-12

**Authors:** Joseph A. Reddy, Ryan Dorton, Alicia Bloomfield, Melissa Nelson, Christina Dircksen, Marilynn Vetzel, Paul Kleindl, Hari Santhapuram, Iontcho R. Vlahov, Christopher P. Leamon

**Affiliations:** Endocyte, Inc., 3000 Kent Ave., Suite A1-100, West Lafayette, IN 47906 USA

## Abstract

EC1456 is a folate-tubulysin conjugate constructed with an all-D enantiomeric spacer/linker configuration. When tested against folate receptor (FR)-positive cells, EC1456 demonstrated dose-responsive activity with an approximate 1000-fold level of specificity. Treatment of nude mice bearing FR-positive human xenografts (as large as 800 mm^3^) with non-toxic doses of EC1456 led to cures in 100% of the mice. Combinations of low dose EC1456 with standard of care agents such as platins, taxanes, topotecan and bevacizumab, safely and significantly augmented the growth inhibitory effects of these commonly used agents. When tested against FR-positive human tumor xenograft models having confirmed resistance to a folate-vinca alkaloid (vintafolide), cisplatin or paclitaxel, EC1456 was found to generate partial to curative responses. Taken together, these studies demonstrate that EC1456 has significant anti-proliferative activity against FR-positive tumors, including models which were anticancer drug resistant, thereby justifying a Phase 1 trial of this agent for the treatment of advanced human cancers.

## Introduction

The folate receptor (FR) is functionally expressed in high quantities by many primary and metastatic cancers^1,2^. The vitamin folic acid has been shown to specifically deliver a wide variety of therapeutic- and imaging-based agents to tumors that express the FR protein^3,4^. Hence, we have been developing folate-targeted small molecule drug conjugates (SMDC’s) to boost the safety and efficacy of oncology agents, resulting in an increased therapeutic advantage^5–12^.

The tubulysin family of secondary metabolites were originally isolated from the myxobacteria *Archangium geophyra* and *Angiococcus disciformis*. A variety of tubulysin analogs^13–17^ have been synthesized and tested for their activity towards different cancers^18–20^. These compounds are potent microtubule destabilizing agents with IC_50_ values in the picomolar range against many cancer cell lines^21,22^, including those with multidrug resistant properties^23^.

In spite of the powerful *in vitro* activity of tubulysins, they have limited *in vivo* therapeutic activity due to severe toxicity. For example, the natural tubulysin B drug in our hands proved to be inactive against a human cervical cancer tumor model when administered at doses near to or greater than the maximum tolerated dose (MTD)^6^. For this reason, we believe that tubulysins are perfect candidates to be incorporated into our SMDC delivery system. We have recently described the biological activity of EC0305^6^ and subsequently EC0531^24^, which are folate conjugates of tubulysin B^12,25^. Here we report on a detailed *in vivo* investigation of a folate tubulysin conjugate (EC1456) that exploits the stable, water soluble saccharo-peptide spacer already tested in EC0531, with particular emphasis on efficacy towards large subcutaneous tumors, combinations with standard of care agents and activity against relevant drug-resistant tumor models.

## Results

### EC1456 is the all-D enantiomer of EC0531

**Figure 1:**
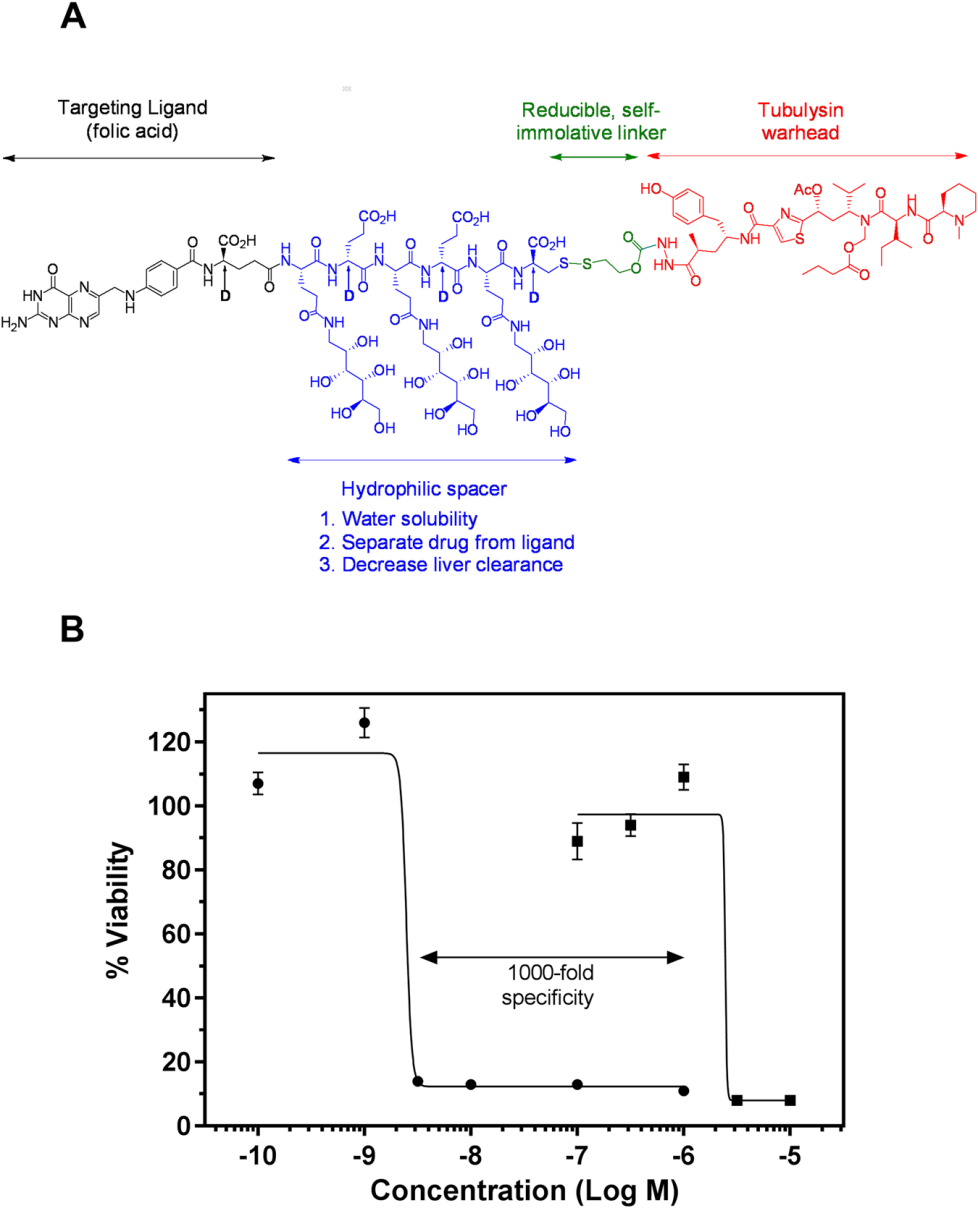
Chemical structure and *in vitro* activity of EC1456. (**A**) Module 1 (in black) is the tumor-targeting ligand, folic acid. Module 2 (in blue) is a hydrophilic saccharo-peptidic spacer. Module 3 (in green) is a bio-cleavable, self-immolative disulfide-based linker system. Module 4 (in red) is the active agent, tubulysin B hydrazide. (**B**) KB cells were pulsed for 2 h with increasing concentrations of EC1456 in the absence (⚫) or presence of 100 µM folic acid (◼) as a benign competitor.

All FA-drug conjugates reported to date contain a modular design^26^. The color-coded modularity of the EC1456 structure is shown in Fig. 1A. This SMDC contains multiple polar carbohydrate segments constructed with novel 1-amino-1-deoxy-glucitolyl-γ-glutamate residues, each separated from the other with d-Glu residues and then terminating with d-Cys. Selection of this saccharopeptidic spacer was based on prior results with other SMDCs showing the need for sterically increasing the hydrophilic spacer region to dis-allow, or significantly reduce non-FR mediated cellular uptake, particularly in highly perfused organs like the liver^10,24^.

### EC1456’s *in vitro* activity is dose-dependent and specific for the FR

As shown in Fig. 1B, FR-positive KB cells were found to be highly sensitive to EC1456 with an IC_50_ of 1.5 nM, a value that is also nearly identical to that measured for its all L-enantiomer, EC0531^24^. This result was important because it confirmed that the activity (i.e. drug release) of a disulfide-based SMDC was not dependent on the stereo-specificity of the spacer-linker moieties. EC1456’s activity was next confirmed to be dependent on FR expression since an excess folic acid (used as a benign competitor ligand) reduced EC1456’s cytotoxicity by ~1000-fold (Fig. 1B).

### Anti-tumor activity of EC1456 against large KB tumor xenografts

**Figure 2:**
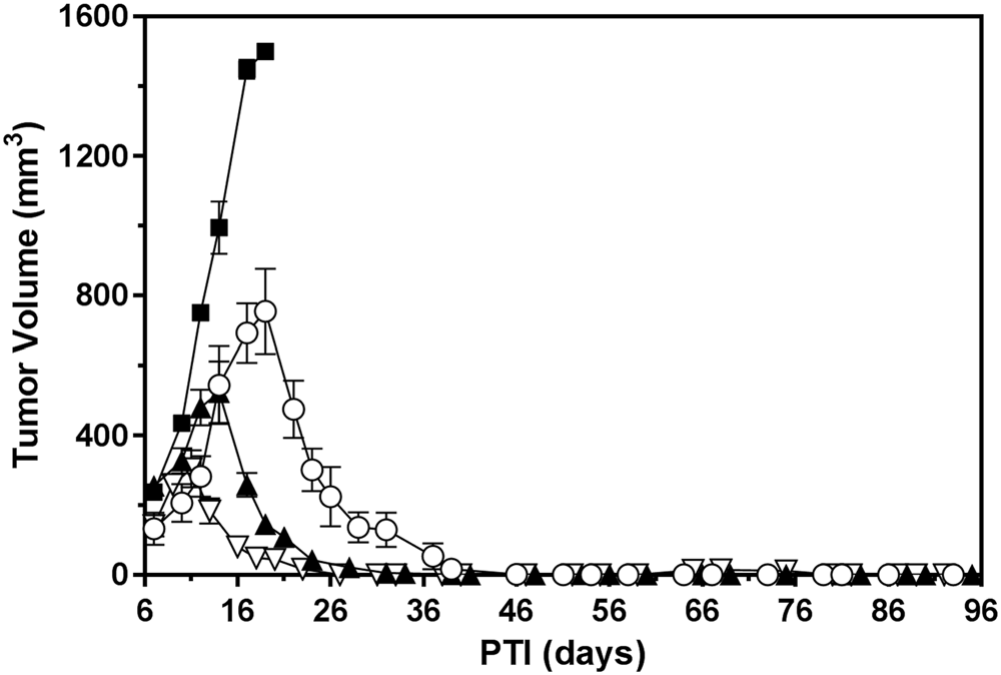
Antitumor effects of EC1456 on various size tumors. KB tumor cells (1 × 10^6^) were inoculated subcutaneously into *nu/nu* mice and randomized with tumors in various ranges. Mice were treated with EC1456, 2 μmol/kg, TIW x 2 weeks. Tumor volume ranges: (▽) 224–312 mm^3^; (▲) 386–617 mm^3^; (○) 640–821 mm^3^. ◼, untreated control cohort. Each curve shows the average volume of 4–5 tumors.

The activity of EC1456 against the FR-positive parental KB tumor model was assessed by treating mice bearing tumors of increasing sizes with 2 μmol/kg at a three times per week (TIW), 2-week schedule. Mice were divided into three groups and treatments started when the tumors had reached the following range: 224–312 mm^3^, 386–617 mm^3^ and 640–821 mm^3^. As shown in Fig. 2, untreated control mice reached a tumor size of 1500 mm^3^ by approximately PTI day 19, whereas treatment with EC1456 lead to 100% cures in all groups, regardless of tumor size at the onset of dosing. Importantly, EC1456-treated animals did not lose any significant weight throughout the dosing period and beyond, which is similar to that seen with our previously reported folate-targeted cytotoxic agents^5–7^.

### EC1456 combines synergistically with various clinical anticancer agents

FR-targeted drugs like vintafolide and EC1456 have or are currently being tested clinically against FR-expressing cancers such as ovarian, non-small lung cancer, endometrial and triple-negative breast cancer. The anticancer agents cisplatin, carboplatin, carboplatin/paclitaxel combination, docetaxel, topotecan and bevacizumab are general treatment options for patients with these cancers. In the following studies, EC1456 was combined with these standard agents and evaluated against the FR-expressing KB tumor model for evidence of combined anti-tumor activity.

All of the drug combinations were tested using a lower, less efficacious 1 μmol/kg EC1456 dose level, following either a twice a week (BIW) × 2 week regimen (A) or a TIW × 2 week regimen (B) (see Table 1). When combined with EC1456 on regimen A the DNA crosslinker, cisplatin, produced 100% cures under conditions where single agent EC1456 produced 0 PRs and cisplatin produced only 60% PRs. Carboplatin also combined very well with EC1456 (regimen B) resulting in 100% cures, while EC1456 and carboplatin alone were less effective. At a lower dose of carboplatin (30 mg/kg) and when combined with paclitaxel, significant antitumor activity (80% CRs/20% cures) was observed. Yet, adding EC1456 to this doublet led to 100% cures with no additional toxicity.

As single agents both the microtubule inhibitor, docetaxel, and EC1456 (regimen B) produced impressive antitumor activity, with 40% cures/60% PRs and 40% cures/60% CRs, respectively. But when combined, EC1456 + docetaxel worked together to cure 100% of the treated animals, again with no significant change in adverse events.

Significantly greater antitumor effect was also observed when EC1456 was combined with the topoisomerase inhibitor, topotecan. As single agents, topotecan and EC1456 (using the less frequent dosing regimen A) produced minimal antitumor activity as noted by 0% PRs and 20% PRs, respectively. In contrast, this EC1456/topotecan combination produced 40% cures/40% CRs/20% PRs.

EC1456 on regimen B also combined very well with a VEGF inhibitor, bevacizumab, yielding 100% cures, whereas bevacizumab alone or single agent EC1456 produced far lower responses, as noted in Table 1.

**Table 1:** Activity and response for each drug combination tested in the KB tumor-*nu/nu* mouse tumor model.

Treatment Regimen	PR, %	CR, %	Cures, %
**Control mice**	0	0	0
**EC1456** (A: 1 μmol/kg BIW × 2)	0	0	0
**Cisplatin** (3 mg/kg BIW 3 doses)	60	0	0
**EC1456** (A: 1 μmol/kg BIW × 2) + **Cisplatin** (3 mg/kg BIW 3 doses)	0	0	100
**Control mice**	0	0	0
**EC1456** (B: 1 μmol/kg TIW × 2)	60	0	0
**Carboplatin** (50 mg/kg TIW)	0	0	0
**EC1456** (B: 1 μmol/kg TIW × 2) + **Carboplatin** (50 mg/kg TIW)	0	0	100
**Control mice**	0	0	0
**EC1456** (A: 1 μmol/kg BIW × 2)	20	0	0
**Carboplatin** (30 mg/kg BIW × 2) + **Paclitaxel** (10 mg/kg BIW × 2)	0	80	20
**EC1456** (A: 1 μmol/kg BIW × 2) +**Carboplatin** (30 mg/kg BIW × 2) +**Paclitaxel** (10 mg/kg BIW × 2)	0	0	100
**Control mice**	0	0	0
**EC1456** (B: 1 μmol/kg TIW × 2)	0	60	40
**Docetaxel** (7 mg/kg BIW 3 doses)	60	0	40
**EC1456** (B: 1 μmol/kg TIW × 2) + **Docetaxel** (7 mg/kg BIW 3 doses)	0	0	100
**Control mice**	0	0	0
**EC1456** (A: 1 μmol/kg BIW × 2)	20	0	0
**Topotecan** (5 mg/kg BIW × 2)	0	0	0
**EC1456** (A: 1 μmol/kg BIW × 2) + **Topotecan** (5 mg/kg BIW × 2)	20	40	40
**Control mice**	0	0	0
**EC1456** (B: 1 μmol/kg TIW × 2)	60	20	20
**Bevacizumab** (5 mg/kg BIW × 2)	0	0	0
**EC1456** (B: 1 μmol/kg TIW × 2) + **Bevacizumab** (5 mg/kg BIW × 2)	0	0	100

All of the drug combinations were tested using a 1 μmol/kg EC1456 dose level, at either a twice a week (BIW) x 2 week regimen (A), or a TIW x 2 week regimen (B). n = 5/cohort. PR, partial response defined as volume regression >50% but with measurable tumor (>2 mm^3^) remaining at all times; CR, complete response defined as a disappearance of measurable tumor mass (<2 mm^3^) at some point within 90 days after tumor implantation; cures were defined as CRs without tumor regrowth within the 90-day study time frame.

### Anti-tumor effects of vintafolide and EC1456 on KB-145-55 tumors

**Figure 3:**
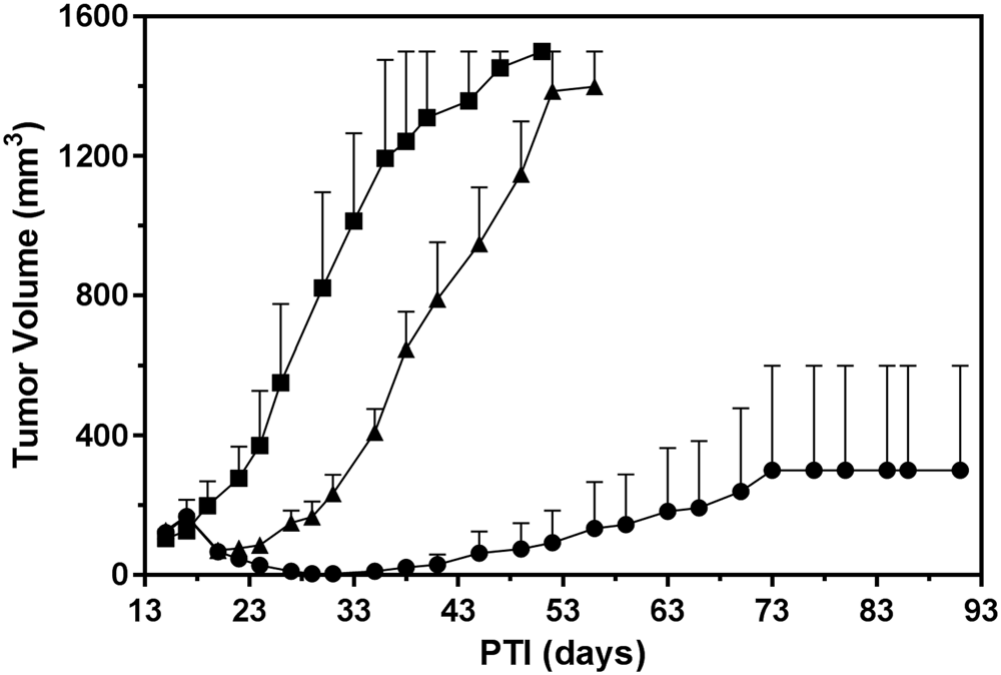
Anti-tumor effects of vintafolide and EC1456 against KB-145–55 tumors. Vintafolide-resistant KB-145–55 tumor cells (1 × 10^6^) were inoculated subcutaneously into *nu/nu* mice and therapy started on randomized animals with tumors in the 93–173 mm^3^ range. ■, Control; ▲, vintafolide, 2 μmol/kg, TIW x 2 weeks; ●, EC1456, 2 μmol/kg, TIW × 2 weeks. The EC1456 treatment (●) curve is the result of re-growth of only 1 of 5 tumors after day 35. Each curve shows the average volume of 5 tumors.

**Figure 4:**
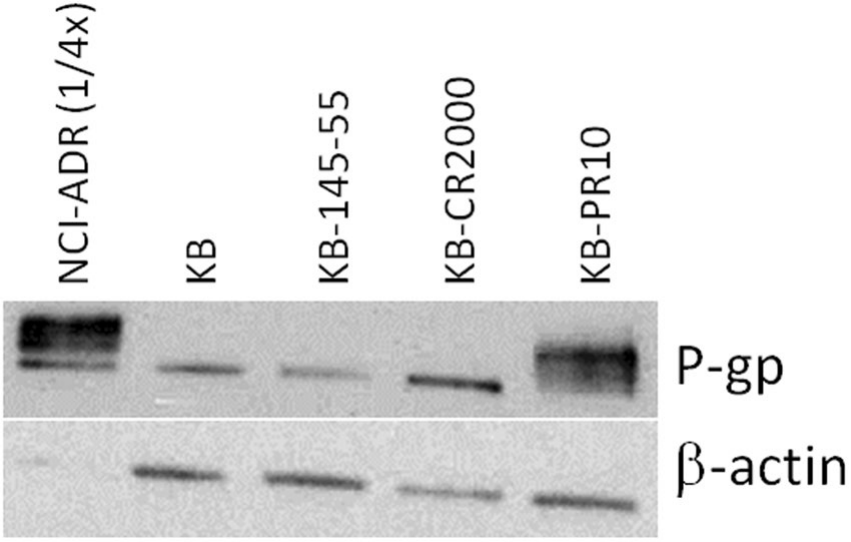
P-glycoprotein (ABCB1, MDR1) expression *in* vintafolide, cisplatin and paclitaxel resistant cell lines. KB, KB-145–55, KB-CR2000, KB-PR10, and NCI-ADR cell lines were lysed with 100 µL RIPA buffer containing 1:100 Halt phosphatase and protease inhibitor cocktail. Ten μg of each protein lysate (2.5 μg of NCI-ADR) were resolved by SDS-PAGE. P-gp and β-actin were detected with rabbit anti-MDR1 (1:1000) and rabbit anti-β-actin (1:2000) antibodies, respectively. A horseradish peroxidase (HRP)-conjugated goat anti-rabbit antibody (1:5000) was used to visualize the signal by an ECL substrate. Western blot image (B) has been cropped for clarity with full blots presented in Figure S1.

The KB-145-55 cell line was created from a parental KB tumor which became resistant to vintafolide (a clinically tested folate-vinca alkaloid conjugate) in a mouse intermittently dosed 43 times with 2 μmol/kg of drug over a 192 day span. The level of KB-145-55’s resistance to vintafolide was assessed by treating the mice 15 days PTI using a normally 100% curative regimen (i.e. 2 μmol/kg given TIW, for 2 weeks;^7^). As expected and shown in Fig. 3, vintafolide produced a greatly reduced level of antitumor activity (zero CRs and three PRs) in these resistant tumors. This compromised efficacy could not be explained by minor changes in FR copies/cell (6.31 × 10^6^ vs 6.33 × 10^6^ receptors/cell for KB-145-55 and parental KB cells, respectively), tumor growth rate (37 vs. 28 days to reach 1500 mm^3^ in volume for KB-145-55 and parental KB cells, respectively), or p-glycoprotein (P-gp) expression levels (Fig. 4). In contrast, EC1456 administered using the same dosing regimen had generated a 100% (5/5) complete response rate, of which four of the five animals maintained these CR’s throughout the 90-day duration of the study and were considered cured.

### Generation of cisplatin and paclitaxel resistant cell lines

Parental KB cells were next used to generate drug-resistant, FR-expressing cell lines through increasing levels of cisplatin or paclitaxel exposure. The cell lines derived through exposure to cisplatin (KB-CR2000, IC_50_ ~ 10 μM) and paclitaxel (KB-PR10, IC_50_ ~ 130 nM) both exhibited significant resistance (Table 2) to their corresponding drugs as compared to the parental KB cells (cisplatin IC_50_ ~ 380 nM, paclitaxel IC_50_ ~ 3 nM).

**Table 2:** *In vitro* activity (IC50 values) of various agents on KB, KB-CR2000 and KB-PR10 cells.

Drug/s	KB	KB-CR2000	KB-PR10
Cisplatin	379 nM	10.8 μM	—
Paclitaxel	3 nM	—	132 nM
Vintafolide	9 nM	5 nM	>1 μM
Paclitaxel + Verapamil	—	—	5 nM
Vintafolide + Verapamil	—	—	<1 nM
EC1456	2 nM	4 nM	2 nM

Analysis by western blotting revealed that P-gp expression in the KB-PR10 cells was greatly amplified (~13 fold) in comparison to the parent KB cells or the KB-CR2000 cells (Fig. 4). NCI-ADR cells, which are a known high P-gp expressing cell line, were used as a positive control, albeit at one fourth of the protein concentration as the other cells. This overexpression of P-gp in KB-PR10 cells also caused them to be cross-resistant (IC_50_ > 1 μM) to vintafolide, which was expected^27^. The low P-gp expressing KB and KB-CR2000 cells remained sensitive (IC_50_ of 5–9 nM) to vintafolide. When combined with either paclitaxel or vintafolide, verapamil (a potent inhibitor of P-gp) treatment (10 μM) had restored KB-PR10’s sensitivity to both drugs (IC_50s_ of 1–5 nM). Verapamil alone at 10 μM showed minimal toxicity on KB-PR cells with 90.8 +/− 5.1% of cells remaining viable compared to untreated controls. However, in stark contrast, EC1456 remained potent in all three cell lines (IC_50_ of 2–4 nM), regardless of their P-gp expression levels (Table 2).

### Anti-tumor effects of cisplatin, vintafolide and EC1456 against KB-CR2000 tumors

**Figure 5:**
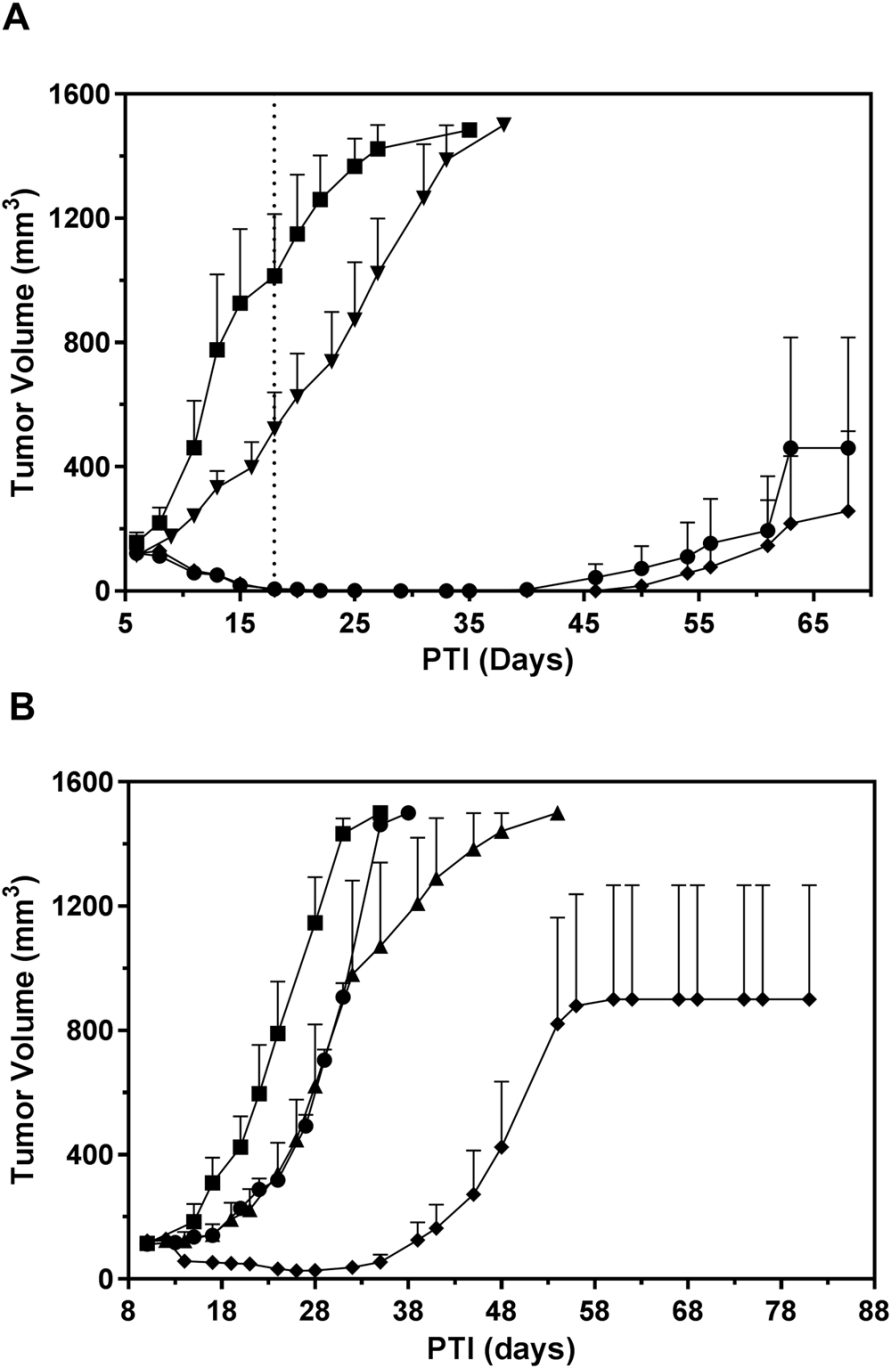
Antitumor effects of cisplatin (**A**)/paclitaxel (**B**), vintafolide and EC1456 against cisplatin-resistant KB-CR2000 tumors (A) and paclitaxel resistant KB-PR10 tumors (**B**). KB-CR2000 (**A**) and KB-PR10 (**B**) tumor cells (1 × 10^6^) were inoculated subcutaneously into *nu/nu* mice and therapy started on randomized animals with tumors in the 99–149 mm^3^ range. ■, Control; ▼ (**A**), cisplatin, 3 mg/kg, BIW x 2 weeks; ▲ (**B**), paclitaxel, 20 mg/kg, TIW x 2 weeks; ●, vintafolide, 2 μmol/kg, TIW x 2 weeks; ◆, EC1456, 2 μmol/kg, TIW x 2 weeks. Each curve shows the average volume of 5 tumors.

To build on the results observed *in vitro*, vintafolide, cisplatin, and EC1456 were dosed in *nu/nu* mice that had been inoculated subcutaneously with KB-CR2000 cells. The *in vivo* activity of these three agents against KB-CR2000 tumors was analogous to the *in vitro* activity of these drugs on KB-CR2000 cells. As expected, cisplatin alone was found to be inactive against the KB-CR2000 tumors (Fig. 5A). However, vintafolide remained highly active against this cisplatin-resistant model yielding 50% CRs/50% cures, and EC1456 at the same dose and schedule produced an even better response with 20% CRs and 80% cures. Changes in FR copies/cell (5.77 × 10^6^ vs. 6.33 × 10^6^ receptors/cell for KB-CR2000 and parental KB cells, respectively) and tumor growth rates (25 vs. 28 days to 1500 mm^3^ for KB-CR2000s and parental KB cells, respectively) were minimal to have significant effects on drug sensitivities of these tumors.

### Anti-tumor effects of paclitaxel, vintafolide and EC1456 on KB-PR10 tumors

The paclitaxel-resistant cells, KB-PR10, were inoculated subcutaneously into mice and treatment started when tumors were in a range of 99–144 mm^3^. As shown in Fig. 5B, both single agent paclitaxel and vintafolide had very little effect against the tumors, with 40% PRs and 0% PRs, respectively. On the other hand, single agent EC1456 substantially reduced tumor burden resulting in 60% PRs and 40% cures. FR copies/cell (7.95 × 10^6^ vs. 6.33 × 10^6^ receptors/cell for KB-PR and parental KB cells, respectively) and tumor growth rates (31 vs. 28 days to 1500 mm^3^ for KB-PR and parental KB cells, respectively) were quite similar to those of KB tumors to have any significant effects on KB-PR drug sensitivities.

## Discussion

EC1456 is the third iteration of a folic acid-based SMDC constructed with the potent tubulysin B hydrazide (TubBH) drug linked together with a disulfide bond. The first folate-tubulysin B conjugate reported by us is EC0305, which was constructed with a γGlu-Arg-Asp-Cys spacer connecting the folate moiety to TubBH^6^. Subsequently, EC0305 was re-engineered with a saccharopeptidic-based hydrophilic spacer (SPS) replacing the peptide spacer. The resulting molecule, called EC0531, thus contained polar carbohydrate (1-amino-1-deoxy-glucitolyl-γ-glutamate) segments, separated by L-Glu residues and terminating with L-Cys^24^. EC0531 was found to be more active over a wider therapeutic range than EC0305, as supported by a 5.6-fold less free TubBH clearing through the bile following EC0531 injection when compared to EC0305. Within EC1456, we retained the positive pharmacokinetic properties of the SPS spacer system while replacing the L-glu and L-cys amino acids with their D-isoforms. Such stereochemical modifications are known to enhance peptidic biostability because of resistance to proteolytic degradation by most of the endogenous enzymes^28^. This change in chirality would also likely limit the pericellular proteolysis of EC1456 by membrane associated and secreted proteases in the tumor^29^.

Following brief cycles of EC1456 therapy, tumors of large sizes were repeatedly observed to totally recede, mostly without recurrence, while producing no noticeable weight loss or adverse events, suggesting that EC1456 may be a useful agent against FR-expressing human cancers. In comparison, untargeted TubBH, regardless of the dose, was not found to produce any antitumor effect in this parental KB tumor model^12^. This anti-tumor effect against very large s.c. tumors is most probably due to homogeneous EC1456 cellular targeting as previously demonstrated by the uniform fluorescence in the center and periphery of tumors in mice dosed with a small molecule folate-rhodamine conjugate^7^. ADCs, in contrast, owing to their slow tissue penetrating abilities, often saturate receptors of perivascular cells causing heterogeneous distribution within solid tumors which can impact their overall efficacy^30^.

Since standard clinical chemotherapy regimens generally use multidrug combinations, we proceeded to determine if EC1456 could improve therapeutic efficacy by combining with clinically approved agents with various mechanisms of action. When low dose EC1456 was combined with the DNA crosslinking agents, cisplatin and carboplatin, enhanced antitumor activity was observed over that produced by the single agents, with some studies yielding 100% cure rates. When EC1456 was added to the often clinically used paclitaxel/carboplatin doublet, it improved the antitumor effect by again yielding 100% cures. EC1456 was also tested in combination with a microtubule inhibitor, docetaxel, the topoisomerase inhibitor, topotecan, and the VEGF inhibitor, bevacizumab. In all cases, the antitumor activity of the EC1456 combinations was far superior to that observed with any of the individual drugs tested alone. In addition, combining EC1456 with these agents did not increase the inherent toxicity above that produced by the untargeted agents, thus resulting in a general increase in the EC1456 combination therapeutic window.

In the past we^6^ and others^23^ have shown that tubulysins retain their activity against multidrug resistant cell lines, while vinca alkaloids as a class do not. Our first generation SMDC, vintafolide, is a folic acid-targeted conjugate of a vinca alkaloid. To test if EC1456 would be useful in treating tumors that have developed resistance toward vintafolide, we created a FR-positive cell line KB-145–55, from a tumor which had been made resistant to vintafolide *in vivo*. We confirmed herein that vintafolide has reduced antitumor activity against KB-145-55 tumors in comparison with the parental KB tumors, whereas EC1456 (dosed similarly) yielded curative activity, likely because of tubulysin being not a good P-gp substrate.

A majority of ovarian cancer patients receive a first-line combination regimen that comprises a taxane and a platinum drug. There is a clear need in advanced ovarian cancer to consider the use of second-line chemotherapeutic options, since most women will ultimately relapse and develop drug-resistant disease^31,32^. To pre-clinically forecast EC1456’s activity in such patients, paclitaxel and cisplatin-resistant variants of FR-expressing KB cells were selected by continuous in *vitro* exposure to increasing concentrations of these drugs. When cross resistance was investigated, it was found that the cisplatin-derived resistant line, KB-CR2000, was not cross-resistant to vintafolide. In contrast, the paclitaxel-derived resistant cells, KB-PR10, exhibited significant cross resistance to vintafolide, but not to the tubulysin-containing EC1456. Not surprisingly, the KB-PR10 subline did exhibit a multidrug-resistance phenotype with overexpression of p-glycoprotein, whereas KB-CR2000 did not. Hence verapamil, a P-gp inhibitor, was able to revive KB-PR10’s sensitivity to both paclitaxel and vintafolide. As would be predicted from the cell culture data, the anti-tumor activity of EC1456 was similar to that of vintafolide in the low P-gp expressing KB-CR2000 model, but EC1456 was found to be far superior to vintafolide against the higher P-gp expressing KB-PR10 tumor model. This outcome indicated that the activity of EC1456 was predominantly independent of the levels of p-glycoprotein expression, thereby confirming the previous findings of tubulysin not being a good substrate of this protein^23^.

While our first generation SMDC, vintafolide, was unable to have much of an effect against some drug-resistant models, the tubulysin-containing EC1456 was able to show curative effects. These results may suggest that EC1456 could be useful in treating tumors that have developed resistance towards some standard chemotherapeutic agents. These exciting preclinical qualities have provided justification for a Phase 1 clinical trial.

## Methods

### Materials

Clinical vial solutions of vintafolide (formally EC145) and EC1456 were used in all experiments (Endocyte Inc.). Carboplatin, cisplatin and verapamil HCl were purchased from Sigma, St. Louis, MO; docetaxel and bevacizumab were obtained from the Purdue University Pharmacy, West Lafayette, IN; paclitaxel and topotecan were purchased from A.K. Scientific, Mountain View, CA. NCI-ADR cells were a kind gift from Dr. Alberto Gabizon, Shaare Zedek Medical Center, Israel. All other common reagents were purchased from Sigma or other major suppliers.

### Chemical characterization of EC1456

^1^H and ^13^C NMR were obtained on an Agilent 500 MHz NMR. All experiments were conducted at 25 °C. All spectra were referenced to the DMSO solvent residual signals at 2.5ppm (^1^H) and 39.50 ppm (^13^C). Positive electrospray mass spectra were obtained on a high resolution Waters Xevo G2-S TOF mass spectrometer following a reverse-phase separation of the major peak on the Acquity UPLC chromatographic system.

HPLC purity: 96% (XBridge RP18 3.5 µm, 3.0 × 50 mm column at λ = 280 nM); ^1^H NMR (500 MHz, DMSO-*d*_6_/D_2_O): *δ* 8.59 (s, 1 H), 8.14 (s, 1 H), 7.56 (d, *J* = 8.5 Hz, 2 H), 6.95 (d, *J* = 8.0 Hz, 2 H), 6.61 (d, *J* = 8.5 Hz, 2 H), 6.57 (d, *J* = 8.0 Hz, 2 H), 6.16 (d, *J* = 9.5 Hz, 1 H), 5.68 (d, *J* = 12.0 Hz, 1 H), 5.22 (d, *J* = 12.0 Hz, 1 H), 4.45 (s, 2 H), 4.37 (d, *J* = 9.0 Hz, 2 H), 4.30–4.0 (m, 10 H), 3.60 (m, 3 H), 3.59-3.50(m, 6 H), 3.44 (m, 3 H), 3.43–3.37 (m, 6 H), 3.18 (m, 4 H), 3.05 (m, 3 H), 2.98–2.86 (m, 3 H), 2.78 (d, *J* = 11.5 Hz, 2 H), 2.68 (m, 2 H), 2.53–2.45 (m, 2 H), 2.40-2.25 (m, 2 H), 2.23-1.98 (series of m, 15 H), 2.06 (s, 3 H), 2.00 (s, 3 H), 1.98-1.62 (series of m, 14 H), 1.62-1.24 (series of m, 9 H), 1.18-1.00 (m, 2 H), 0.98 (d, *J* = 6.5 Hz, 3 H), 0.92 (d, *J* = 6.0 Hz, 3 H), 0.78 (d, *J* = 7.0 Hz, 3 H), 0.74 (d, *J* = 8.5 Hz, 3 H), 0.73 (d, *J* = 7.5 Hz, 3 H), 0.62 (d, *J* = 6.0 Hz, 3 H); ^13^C NMR (125 MHz, DMSO-d_6_/D_2_O): *δ* 176.77, 176.32, 175.74, 175.42, 174.75, 173.87, 172.68, 172.15, 171.94, 171.84, 173.43, 173.30, 172.79 (2 C), 172.72, 172.46, 170.87, 170.39, 169,30, 166.09, 162.40, 160.70, 156.40, 156.09, 155.71, 154.59, 150.84, 149.63, 149.11, 148.99, 130.44 (2 C), 128.99 (2 C), 128.89, 127.99, 124.97, 122.24, 115.25 (2 C), 111.86 (2 C), 72.17 (3 C), 71.78, 71.74, 71.71, 71.62, 71.59 (2 C), 69.65, 69.57 (2 C), 69.45, 69.34, 68.51, 63.42 (3 C), 63.03, 55.08, 54.05, 53.88, 53.46 (2 C), 53.33, 52.96 (2 C), 52.89, 52.55, 49.77, 46.07, 44.02, 42.85, 42.34 (2 C), 42.29, 39.52, 38.95, 37.43, 35.95, 35.43, 35.38, 34.86, 32.56, 32.36, 32.16, 32.09 (2 C), 31.81, 30.50, 29.95, 28.60, 28.04, 27.78 (2 C), 27.66, 27.00, 25.01, 24.43, 23.04, 20.86, 20.56, 19.64, 18.36, 18.04, 15.64, 13.72, 10.28; HRMS (ESI) (m/z): [M + H]^+^ calcd. for C_110_H_165_N_23_O_45_S_3_, 2625.81; found, 2626.06.

### Dose-Dependent FR-Specific Activity of EC1456

Parental KB cells (a human cell line from ATCC containing markers of HeLa cervical cancer origin) were seeded in individual 12-well Falcon plates and allowed to form nearly confluent monolayers overnight in folate-deficient RPMI medium supplemented with 10% fetal bovine serum. Following a detailed published procedure^12^, a 2 h pulse, 70 h chase assay format was used to evaluate the cytotoxic effects of increasing concentrations of EC1456. Viability was assessed by measuring ^3^H-thymidine incorporation into trichloroacetic acid precipitable material. Final results were expressed as the percentage of ^3^H-thymidine incorporation relative to untreated controls.

### Creation of an *in vivo*, vintafolide-resistant EC145-55 model

FR-positive human nasopharyngeal carcinoma KB cells (6.0 × 10^6^ FRs/cell) were used in this study; their integrity was confirmed (Genetica DNA Labs, Cincinnati, Ohio) with cells being similar to reference cells from the ATCC.

A vintafolide-resistant KB tumor model was created after continuous controlled treatment *in vivo*. For this, ten 6-7 week-old female *nu/nu* mice maintained on a custom folate deficient diet were each subcutaneously inoculated with 1 × 10^6^ KB cells. These tumors were allowed to grow to 250 mm^3^ after which these mice were dosed with 2 µmol/kg vintafolide. The tumors were measured three times a week and the decision to dose was based on the tumor volume. This controlled dosing method was used such that the tumor volumes were maintained between 100 mm^3^ and 300 mm^3^ as long as possible through intermittent dosing (*vide infra*). Although several tumors showed resistance to vintafolide, one tumor-bearing mouse, herein referred to as EC145-55, received a total of 43 vintafolide doses. The mouse bearing the EC145-55 tumor received its first dose of vintafolide on PTI (post tumor cell implant) day 17 when the tumor was 272 mm^3^. The tumor volume oscillation process was maintained by dosing on PTI days 19, 45, 47, 68, 78, 85, 94, 96, 109, 113, 115, 117, 127, 129, 141, 143, 148, 150, 152, 155, 157, 159, 162, 164, 166, 171,173,176, 178, 180, 183, 185, 187, 190, 192, 194, 197, 199, 204, 206, 208, and 211. Following the 43^rd^ dose on PTI day 211, the mouse was euthanized, tumor extracted at a volume of 671 mm^3^, manually disaggregated and then placed into cell culture for further growth and analysis.

### Generation of KB-CR2000 and KB-PR10 cells

Our drug-sensitive FR-expressing KB cell line was used as a parental line to generate additional drug-resistant cell lines through increasing levels of cisplatin and paclitaxel exposure. Cisplatin and paclitaxel stock solutions were made in saline and DMSO respectively; appropriate volumes of the sterile stock solutions were added to cell culture flasks to achieve the desired concentration of drug supplemented media. Drug concentrations were increased when cell death was no longer visible and growth rate had stabilized. Thus, cisplatin-resistant KB cells (KB-CR2000) were selected *in vitro* through exposure to stepwise-increasing concentrations of cisplatin from 50 to 2000 nM, while paclitaxel-resistant KB cells (KB-PR10) were selected with concentrations from 0.1 to 10 nM paclitaxel, with 0.5- to 1-fold increase at each step of resistance.

### Cell growth inhibition studies

Parental KB cells, KB-CR2000 and KB-PR10 cells were maintained in folate-free RPMI medium (FFRPMI) containing 10% heat-inactivated fetal calf serum (HIFCS) at 37 °C in a 5% CO_2_/95% air-humidified atmosphere with no antibiotics. Exponentially growing cells were seeded in 24-well plates 24 h before treatment with drugs. Cells receiving FR-targeted drugs (vintafolide and EC1456) were pulsed for 2 h at 37 °C, rinsed 4 times with 0.5 mL of medium, and then chased in 1 mL of fresh medium for up to 72 h. Cells treated with non-targeted drugs (cisplatin, paclitaxel and verapamil) were treated continuously for 72 h. Cells were treated with fresh medium containing ^3^H-thymidine for 2 h at 37 °C, washed with phosphate-buffered saline (PBS) and then treated with ice-cold 5% trichloroacetic acid (TCA). After 15 min, the TCA was aspirated and cells solubilized by the addition of 0.25 N sodium hydroxide for 15 min at room temperature. Each solubilized sample was transferred to scintillation vials containing EcoLume™ scintillation cocktail and counted in a liquid scintillation counter.

### ***In vivo*antitumor experiments**

Four- to eight-week-old female *nu/nu* mice (Harlan Sprague Dawley, Inc.), were maintained on a standard 12-h light-dark cycle and fed *ad libitum* with a low-folate chow (Harlan Teklad diet #TD.01013, Madison, WI) for the duration of dosing and 1 week post dosing schedule. Mice were then switched to Teklad Global 18% Rodent diet (Harlan Teklad diet #2018S) during the monitoring phase of the study. Parental KB, KB-145-55, KB-CR2000 or KB-PR10 cells (1 × 10^6^ per *nu/nu* mouse) in 100 µL were injected into the subcutis of the dorsal medial area. Mice were divided into groups of 5, and freshly prepared test articles were injected through the lateral tail vein under sterile conditions in a volume of 200 μL of PBS. Intravenous treatments typically initiated on day 15 PTI when KB-145-55 tumors were approximately 93 to 173 mm^3^ in volume, on day 10 PTI when the KB-PR10 tumors were about 96 to 162 mm^3^, and on day 6 PTI when the KB-CR2000 were around 99 to 149 mm^3^. In combination experiments, EC1456 was administered 2-3 h ahead of the combined drug, when given on the same day.

The mice in the control groups received no treatment. Growth of each subcutaneous tumor was followed by measuring the tumor 3 times per week during treatment and twice per week thereafter, until a maximum volume of 1500 mm^3^ was reached. Tumors were measured in 2 perpendicular directions using Vernier calipers, and their volumes were calculated as V = 0.5 × L × W^2^, where L = measurement of longest axis in mm and W = measurement of axis perpendicular to L in mm. As a general measure of gross toxicity, changes in body weights were determined on the same schedule as tumor volume measurements. Survival of animals was monitored daily. Animals that were moribund (or unable to reach food or water) were euthanized by CO_2_ asphyxiation. All animal housing, care, and procedures were followed according to Purdue Animal Care and Use Committee (PACUC)-approved animal care and use protocols.

Individual tumor response endpoints were reported in terms of tumor volume change. A partial response (PR) was defined as volume regression >50% but with measurable tumor (>2 mm^3^) remaining at all times. Complete response (CR) was defined as a disappearance of measurable tumor mass (<2 mm^3^) at some point within 90 days after tumor implantation. Cures were defined as CRs without tumor regrowth within the 90-day study time frame.

### Analysis of P-gp expression

Confluent parental KB, KB-145-55, KB-CR2000, KB-PR10, and NCI-ADR cell lines were cultured in 6 well plates in FFRPMI-1640 with 5% HIFCS with their respective drug concentration, if required (KB-CR2000: 2000 nM cisplatin, KB-PR10: 10 nM paclitaxel), for 24 h. The cells were lysed with 100 µL RIPA buffer containing 1:100 Halt phosphatase and protease inhibitor cocktail for 5 minutes at room temperature with gentle agitation. Protein concentrations were determined by BCA assay, and 10 μg of lysate were resolved by SDS-PAGE. P-gp and β-actin were detected with rabbit anti-MDR1 antibody (1:1000, Cell Signaling) and rabbit anti-β-actin (1:2000, Rockland Immunochemicals), respectively. A horseradish peroxidase (HRP)-conjugated goat anti-rabbit antibody (1:5000, Jackson Immunoresearch) was used to visualize the signal by an ECL substrate (Pierce). Quantitative comparison of pgp expression in these cell lines was done by determining the intensities of the pgp bands with ImageJ.

### Data Availability

The datasets generated during the current study are available from the corresponding author on reasonable request.

## Electronic supplementary material

Supplementary information
