# Supplementary material for: Pre-clinical evaluation of EC1456, a folate-tubulysin anti-cancer therapeutic
Source: Sci Rep. 2018 Jun 12;8:8943. doi: 10.1038/s41598-018-27320-5 (PMC5997627; doi:10.1038/s41598-018-27320-5)
Supplement: Supplementary file 1 — Supplementary information [file 41598_2018_27320_MOESM1_ESM.pdf]

Supplementary Information:

Pre-clinical evaluation of EC1456, a folate-tubulysin anti-cancer therapeutic

Joseph A. Reddy, Ryan Dorton, Alicia Bloomfield, Melissa Nelson, Christina Dircksen, Marilynn Vetzel, Paul Kleindl, Hari Santhapuram Iontcho R. Vlahov and Christopher P. Leamon

Supplementary Figure S1-Full blots for Figure 4B

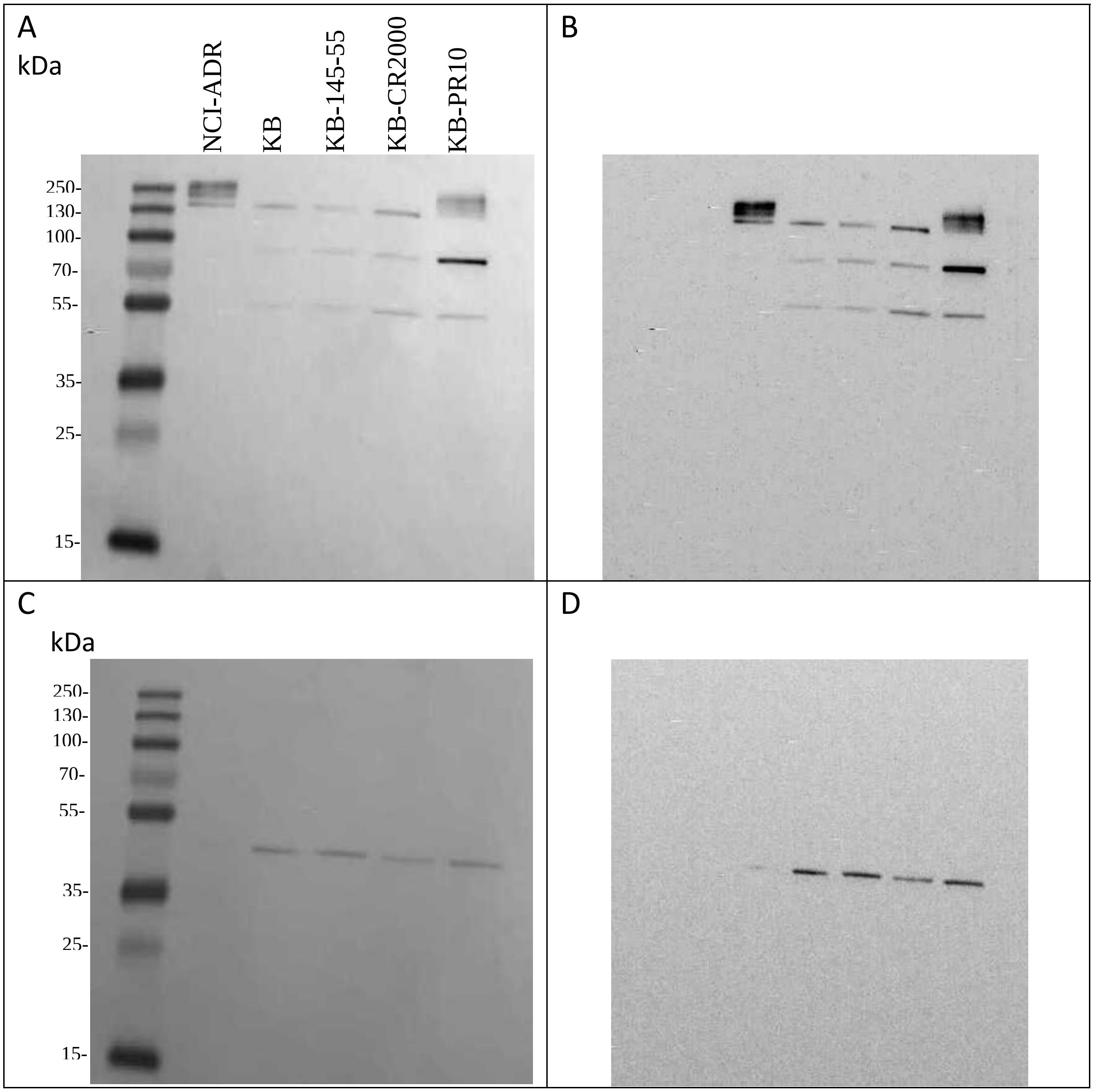

Figure S1. P-glycoprotein (ABCB1, MDR1) expression in vintafolide, cisplatin and paclitaxel resistant cell lines.

KB, KB-145-55, KB-CR2000, KB-PR10, and NCI-ADR cell lines were lysed with 100 µL RIPA buffer containing 1:100 Halt phosphatase and protease inhibitor cocktail. Ten µg of each protein lysate were resolved by SDS-PAGE along with PagerRuler Plus prestained protein ladder. P-gp (A, B) and β-actin (C,D) were detected with rabbit anti-MDR1 (1:1000) and rabbit anti-β-actin (1:2000) antibodies, respectively. A horseradish peroxidase (HRP)-conjugated goat anti-rabbit antibody (1:5000) was used to visualize the signal by an ECL substrate.
